# Clinical and Metabolic Effects of Alpha-Lipoic Acid Associated with Two Different Doses of Myo-Inositol in Women with Polycystic Ovary Syndrome

**DOI:** 10.1155/2020/2901393

**Published:** 2020-03-19

**Authors:** Franca Fruzzetti, Elena Benelli, Tiziana Fidecicchi, Massimo Tonacchera

**Affiliations:** ^1^Department of Obstetrics and Gynecology, Pisa University Hospital, Pisa, Italy; ^2^Department of Endocrinology, Pisa University Hospital, Pisa, Italy

## Abstract

The aim of this retrospective study was to evaluate the effects of a treatment with *α*-lipoic acid (ALA) associated with two different doses of myo-inositol (MI) on clinical and metabolic features of women with polycystic ovary syndrome (PCOS). Eighty-eight women received the treatment, and 71 among them had complete clinical charts and were considered eligible for this study. All women were treated with 800 mg of ALA per day: 43 patients received 2000 mg of MI and 28 received 1000 mg of MI per day. Menstrual cyclicity, BMI, FSH, LH, estradiol, testosterone, androstenedione, fasting insulin, HOMA-IR, and insulin response to a 2 h OGTT were evaluated before and after 6 months of treatment. The presence of diabetic relatives (DRs) was investigated. Cycle regularity was improved in 71.2% of women. The improvement of menstrual cyclicity occurred regardless of the state of IR and the presence of DRs of the patients. Women with IR mainly showed a significant improvement of metabolic parameters, while those without IR had significant changes of reproductive hormones. Patients with DRs did not show significant changes after the treatment. 85.7% of women taking 2000 mg of MI reported a higher improvement of menstrual regularity than those taking 1000 mg of MI (50%; *p* < 0.01). In conclusion, ALA + MI positively affects the menstrual regularity of women with PCOS, regardless of their metabolic phenotype, with a more evident effect with a higher dose of MI. This effect seems to be insulin independent. The presence of IR seems to be a predictor of responsivity to the treatment in terms of an improvement of the metabolic profile.

## 1. Introduction

Polycystic ovary syndrome (PCOS) is a very common endocrine disease of the reproductive age that is defined by the modified Rotterdam criteria of 2003 as the presence of at least two of the following: clinical or biochemical signs of hyperandrogenism, chronic anovulation, and polycystic ovary morphology [1]. Beside these criteria, the metabolic pattern of women with PCOS is a very important feature of the syndrome [2, 3]. Considering the pivotal role of hyperinsulinemia and insulin resistance (IR) in the pathogenesis of PCOS [4], insulin sensitizers have been proposed for the management of these patients [5, 6].

Inositols are involved in the postreceptor signal transmission of several receptors, such as insulin, follicle-stimulating hormone (FSH), and thyroid-stimulating hormone (TSH), and myo-inositol (MI) is one of the most commonly used isoforms of inositol [7, 8]. MI can be incorporated in the inositol phosphoglycan (IPG), a membrane phospholipid that is involved in insulin signal transduction. Insulin interaction with its receptor can activate this transduction pathway mediated by inositols, bringing the constitution of intracellular messengers that are involved in glucose oxidative metabolism instead of nonoxidative metabolism. The MI-IPG can reduce IR and improve glucose metabolism [9]. In fact, it regulates the translocation of GLUT4 to the cellular membrane, and it downregulates the release of free fatty acids by modulating the enzyme adenylate cyclase [10].

Normally, MI is enzymatically converted into another important inositol, D-chiro-inositol (DCI), by an epimerase stimulated by insulin [11]. In PCOS women with IR, the epimerase activity is dysregulated, causing an alteration of the normal balance of these two isomers both in plasma and in peripheral tissues [12]. As a result, the altered balance of inositols in PCOS patients might contribute to both IR and reproductive problems [12–14]. Many studies have been performed to assess the efficacy of MI in improving insulin sensitivity and ovarian function in women with PCOS and IR [8, 15–17]. MI supplementation at the dose of 2–4 g has shown to be effective in ameliorating both metabolic and reproductive features in PCOS women, reducing insulin plasma levels and IR, and improving the oocyte quality and menstrual cycle [8, 18–21].

In very recent times, *α*-lipoic acid (ALA) has been considered a possible therapeutic approach to PCOS and IR [22, 23]. ALA and its reduced form, dihydro-lipoic acid (DHLA), are powerful antioxidant molecules that can act as a scavenger of the reactive oxygen species (ROS) and can regenerate other antioxidant molecules [24]. Moreover, ALA is an inhibitor of the inflammatory pattern mediated by the nuclear factor kappa-light-chain-enhancer of activated B cells (NF-*κ*B) [25], and it also has an immunomodulatory function [26].

In the metabolic field, ALA can improve insulin sensitivity through the activation of the expression of *5′-adenosine monophosphate-activated protein kinase* (AMPK), a cellular energy sensor that induces the translocation of GLUT4 (glucose transporter 4) to the plasma membrane with an insulin-independent mechanism [27–30]. A reduced ALA synthesis, probably due to the downregulation of the lipoic acid synthase (LASY) that occurs during diabetes mellitus (DM) and IR, is supposed to affect the normal glucose uptake and utilization in skeletal muscle cells [31]. In one study performed on lean, nondiabetic PCOS women, Masharani et al. demonstrated that 1200 mg/die of ALA could improve insulin sensitivity and other metabolic features [32]. The hypothesis is that ALA and MI may potentiate each other in improving IR and then the clinical features of PCOS women (menstrual cyclicity/ovarian function).

Nowadays, only few studies investigated the effects of a combined approach with ALA and MI on women with PCOS, and even less is known about how IR and the presence of familiarity for DM affect the results of the treatment [33–36]. The majority of them were performed administering 800 mg of ALA and 2000 mg of MI daily [33, 34, 36], but some studies used half the dose of MI [35, 37]. No comparative studies have been performed to understand which dose works better in improving the clinical and metabolic features of PCOS women.

Considering the described biological effects of MI and ALA, we may hypothesize that higher doses of MI may be able to improve the effect of ALA and that this combination of molecules could bring better results especially in those women with a higher impairment of insulin metabolism.

This study aims to enlarge the actual knowledge about the efficacy of ALA in PCOS women when associated with MI. First, we studied the changes of reproductive, androgenic, and metabolic parameters of PCOS women after 6 months of treatment with 800 mg of ALA per day combined with MI, subsequently evaluating if the presence of IR and/or of familiarity for type 2 diabetes mellitus influenced the results. Then, we investigated if the same dose of ALA (800 mg) elicits different results when associated with different doses of MI (1000 mg or 2000 mg per day).

## 2. Materials and Methods

In this retrospective study, subjects were selected among patients referred to the Department of Clinical and Experimental Medicine, Sections of Gynaecological Endocrinology and of Endocrinology of the University of Pisa. This study was approved by the local ethical committee (No. 4268).

All the subjects considered had a diagnosis of PCOS according to the Rotterdam criteria [1]. Women with hyperprolactinemia, hypo- or hyperthyroidism, congenital adrenal hyperplasia, Cushing's syndrome, or androgen-secreting tumours were excluded from this study. Women gave their informed consent to drug prescription and data collection and for the use of their anonymous data for clinical publication.

All women received a treatment with ALA and MI (Sinopol®, Laborest S. r. l., Italy) for 6 months and were studied before and after the drug intake. Eighty-eight women who received the prescription were initially selected, and 17 patients were dropped out from this study: 8 women were not compliant with the treatment and 9 women did not provide complete data. Seventy-one patients out of the 88 were considered eligible for this study. All women were treated with the same dose of ALA (800 mg per day). Among them, 43 patients (group A) received 2000 mg of MI per day, while 28 (group B) received 1000 mg of MI per day. Both the formulations of ALA + MI were divided into two oral administrations per day.

All women were asked if they had diabetic relatives (DRs). Body mass index (BMI) (kg/m^2^) was calculated for all 71 women before and after the treatment. Blood samples for the laboratory tests were taken once before starting the treatment and once after 6 months of treatment, and all the samples were immediately analysed. Plasma levels of FSH (mIU/mL), luteinizing hormone (LH) (mIU/mL), estradiol (E_2_) (pmol/L), total testosterone (T) (nmol/L), and androstenedione (A) (nmol/L) were determined in the follicular phase. A 2 h oral glucose tolerance test (OGTT) was performed to assess glucose and insulin concentrations. Insulin response was expressed as the area under the curve (AUC), calculated using the trapezoidal rule and expressed as pmol/L × 120 min. As an indicator of insulin resistance, the homeostasis model assessment of insulin resistance (HOMA-IR) was calculated [38]. A cutoff of 2.5 was used to assess the presence of IR. Forty women had complete laboratory parameters both before and after 6 months of treatment.

Plasma LH, FSH, and E_2_ concentrations were determined by immunometric assays (Johnson & Johnson S. p. A-Ortho Clinical Inc., Rochester, NY). Plasma levels of A were determined by using a radioimmunoassay (Biosource Europe S. A., Nivelles, Belgium). The intra-assay and interassay coefficient of variation (CV) for the A assay was 3.2% to 4.5% and 5.9% to 9.0%, respectively. T concentrations were determined by using a competitive immunoassay (Johnson & Johnson S. p. A-Ortho Clinical Inc.). The intra-assay and interassay CV of T was 2.3% to 3.1% and 4.9% to 7.0%, respectively. Insulin was determined by an immunoradiometric assay (DiaSorin S. p. A., Vercelli, Italy). The intra-assay and interassay CV for the insulin assay was 2.1% to 2.6% and 2.9% to 4.7%, respectively. Glucose levels were assessed by enzymatic methods (Roche Diagnostics, Basel, Switzerland).

All women were asked about their menstrual cyclicity before and after the treatment. Women who basally reported the presence of oligomenorrhea were asked if they had an improvement or no change of cycle length after the treatment. Women who reported hirsutism at the baseline were asked if there was no change, improvement, or worsening of it after the treatment. The patients were submitted to a pelvic transabdominal or transvaginal ultrasound before and after 6 months of treatment, and ovarian morphology (reported as normal or PCO-like [1]) was evaluated.

Seventeen women had IR before the treatment, 49 women had an HOMA-IR < 2.5, and 5 did not have a basal OGTT. Twenty-six women were reported to have one or more DRs. Forty-five women did not have familiarity for type 2 diabetes mellitus. Considering their BMI, 30 women were normal weight, 24 were overweight, and 17 were obese.

Thirty healthy subjects with normal cycles and no symptoms of hyperandrogenism were included as controls for baseline characteristics.

### 2.1. Statistical Analysis

Continuous variables were reported as the mean ± standard deviation (SD), while nominal variables were reported as percentages (%). The differences between the group of patients and the controls at the baseline were calculated using Student's *t*-test for unpaired data. The Shapiro–Wilk test was used to test normality. To evaluate the effect of the treatment, Student's *t*-test for paired data or the Wilcoxon signed-rank test was used, as appropriate. The differences in the effects on the menstrual cycle between the subgroups of patients were tested with the *χ*^2^ test. For all the analysis, a value of *p* < 0.05 was considered statistically significant. IBM® SPSS Statistics® software, version 25, was used for the statistical analysis.

## 3. Results

### 3.1. Baseline Characteristics

The characteristics of the patients and the controls at the baseline are reported in Table 1. Patients with PCOS had higher androgen levels than controls, and reproductive and metabolic parameters were generally compromised. Before the treatment, 59 patients (83.1%) had oligomenorrhea, 46 (64.8%) had hirsutism, and 47 (66.2%) had PCO-like ovaries.

Only 17 women (23.9%) had IR before the treatment. Twenty-six women (36.6%) were reported to have one or more DRs.

## 4. Results of the Treatment

The results of the treatment in the entire group of women are reported in Table 1. BMI was significantly reduced (*p* < 0.05). Moreover, significant results were highlighted only in reproductive parameters, with a reduction of FSH (*p* < 0.01) and an increase of E_2_ (*p* < 0.01). Among the 59 women with oligomenorrhea, 71.2% reported a relevant improvement of menstrual regularity, with a shortening of the menstrual length, while 28.8% reported no change of the cycle after the treatment. 50% of women reported no change of their hirsutism, 39.1% reported an improvement, and 10.9% reported a worsening of it. Only 19.2% normalized their ovarian morphology after the treatment.

**Figure 1:**
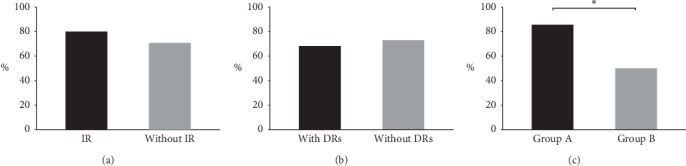
Percentages of women who reported an improvement of menstrual regularity after the treatment. (a) Women with insulin resistance (IR) reported similar changes of menstrual cyclicity than those without IR. (b) Women with diabetic relatives (DRs) reported similar changes of menstrual cyclicity than those without DRs. (c) A higher percentage of women of group A (2000 mg of MI + 800 mg of ALA) reported an improvement of menstrual cyclicity than those of group B (1000 mg of MI + 800 mg of ALA). ^*∗*^*p* < 0.01 (group A vs. group B).

Table 2 summarizes the results obtained dividing patients according to the presence of IR and of DRs. Women with IR showed a significant reduction of BMI (*p* < 0.05) and an increase of E_2_ (*p* < 0.05). Moreover, they showed a relevant improvement of the metabolic pattern, with a reduction of fasting insulin and of HOMA-IR (*p* < 0.01): 80% of them had a normal HOMA-IR after the treatment. Women without IR only showed a significant reduction of FSH (*p* < 0.01) and an increase of E_2_ (*p* < 0.05). Cycle length was improved in 80.0% of patients with IR and in 70.8% of those without IR (*p*=NS) (Figure 1).

DRs did not significantly influence the results. On the contrary, women without DRs showed a relevant improvement of reproductive parameters (FSH, LH, and E_2_), while BMI (*p*=0.06) and HOMA-IR (*p*=0.052) only showed a tendency to a reduction (Table 2). Both the groups of women reported similar results on menstrual regularity, with a reduction of cycle length in 73% of women without DRs and in 68.2% of women with DRs (*p*=NS) (Figure 1).

Table 3 summarizes the results obtained dividing the patients according to the different doses of MI that they received in combination with the same dose of ALA. Group A (800 mg ALA + 2000 mg MI per day) showed a significant change of BMI (*p* < 0.01) and E_2_ and AUC-insulin (*p* < 0.05) after 6 months of treatment, while group B (800 mg ALA + 1000 mg MI per day) showed a significant change of FSH (*p* < 0.01) and LH and E_2_ (*p* < 0.05) but no changes in the metabolic parameters. Cycle length was improved in 85.7% of patients in group A and in 50% of those in group B (*p* < 0.01) (Figure 1).

## 5. Discussion

This study shows the ability of a combination of ALA and MI to restore a normal menstrual cyclicity in women with PCOS, acting on hormonal or on metabolic parameters. The better results were obtained when ALA was associated with a higher dose of MI.

We observed that the improvement of menstrual cyclicity occurs regardless of the state of IR and of the presence of DRs of the patients: every subgroup showed a similar percentage of women (between 68 and 80%) that reported a better condition after 6 months of treatment. Comparing women with and without IR, we found that menstrual cyclicity was restored in both groups, but IR women only also showed an improvement of metabolic parameters. On the contrary, hormonal parameters (FSH and E_2_) seem to be affected by the treatment only in women without IR.

The results obtained in not-IR women are similar to those of De Cicco et al. They studied a group of obese PCOS women without IR treated with MI and ALA for six months, and they found an improvement of cycle length, BMI, hyperandrogenism, and ovarian volume without effects on HOMA-IR and AUC-insulin (both in a normal range at the baseline).

A mandatory role in the improvement of menstrual cyclicity seems to be exerted by the presence of MI in the association. The hypothesis that the effects are mainly MI-mediated is supported by the fact that the only difference in the improvement of menstrual cyclicity was found comparing women who took 2000 mg of MI with those taking 1000 mg of MI: a higher dose of MI associated with the same dose of ALA demonstrated to be more effective in the regularization of the menstrual cycle than the administration of the ALA itself. Anyway, we found also that a low dose of MI can be sufficient to improve the menstrual cyclicity in 50% of women, so maybe this feature is very sensitive to the administration of inositol. Considering the results in women without IR, we can hypothesize that MI can act independently from the IR state. In fact, MI has been recognized to directly facilitate the activity of FSH, acting as a second messenger also for this hormone's receptor [39, 40]. Consequently, in women without IR, MI may be able to act improving FSH response in granulosa cells, thus promoting the normal maturation of the follicle and contributing to normalizing E_2_ levels.

The mechanism through which ALA may have a role in the normalization of the ovarian function is less clear. This is supported also by the results of other studies in the literature. Genazzani et al. in 2018 [41], and again in 2019 [37], demonstrated that ALA alone is not able to affect the reproductive features of PCOS women. In particular, it is difficult to explain which could be its exact role in the improvement of menstrual cyclicity in women without IR. The mechanism seems to be insulin-independent. However, it cannot be excluded that ALA, thanks to its biological activity, may participate in the restoration of the wellbeing of the ovary with an anti-inflammatory action. PCOS is associated with decreased antioxidant concentrations, and it can be considered an oxidative state [42]. WNT5a, a proinflammatory marker, is increased in granulosa cells of both lean and obese PCOS women, and it contributes to the chronic inflammation and to the production of ROS through the activation of the expression of NF-*κ*B [43]. ALA can modulate the NF-*κ*B expression [25]. It is possible that ALA could reduce inflammation and oxidative stress also in the ovary. We may speculate that ALA might contribute to the restoration of a normal environment in the ovary increasing the positive effect of MI.

Women with IR experienced a completely different response to the same treatment. When IR was present, the treatment with ALA and MI reduced fasting insulin and HOMA-IR, restoring a normal insulin sensitivity in almost 80% of the women. This may be due to a synergistic effect displayed by the two insulin-sensitizing molecules: ALA increases the translocation of GLUT4 to the membrane in an insulin-independent way, while MI acts as a second messenger in the pathway of the insulin receptor. When IR is present, high insulin levels impair the normal secretion of LH and FSH from pituitary cells and their function in the ovaries, promoting premature luteinization of follicles [4, 44] Although their effects on gonadotropins and on inflammation, the ability of ALA and MI to restore normal levels of insulin in PCOS women with IR may be another mechanism and probably the condition *conditio sine qua non*, by which these molecules are able to improve menstrual regularity in women with IR.

The clinical efficacy of ALA and MI was not influenced by the presence of DRs. Menstrual cyclicity was similarly improved in both groups, but only women without DRs showed positive results on the reproductive parameters. These results are in contrast with those obtained by other authors, who hypothesized that women with PCOS and DRs had a defect of the LASY similarly to overt diabetes [35, 41]. Therefore, more accurate studies should be performed to understand which alterations are present in PCOS women with DRs, in order to customize the treatment used. Our results suggest that the defect of the LASY is not the only alteration present in this group of women: other mechanisms should be involved, and ALA seems not to be sufficient to act against all of them.

In conclusion, our study demonstrated that the association of ALA and MI can positively affect the menstrual regularity of women with PCOS, with a more evident effect with a higher dose of MI. The presence of IR seems to be a predictor of responsivity to the treatment in terms of an improvement of the metabolic profile, but not in terms of menstrual cyclicity. At present, no explanation seems to be exhaustive, and more studies should be needed to better investigate the mechanisms through which ALA and MI exert their action in women with and without IR.

## Figures and Tables

**Table 1:** Characteristics of the entire group of women at the baseline and after six months of treatment with ALA plus MI.

	Controls	ALA + MI	
		Baseline	6 months
Age (years)	23.1 ± 5.4	21.56 ± 4.77	—
BMI (kg/m^2^)	27.17 ± 3.93	26.97 ± 5.15	26.47 ± 4.95^*∗*^
FSH (mIU/mL)	4.17 ± 0.24	6.86 ± 3.05^b^	5.19 ± 2.44^*∗∗*^
LH (mIU/mL)	4.52 ± 1.49	12.55 ± 7.16^b^	10.26 ± 6.79
Estradiol (pmol/L)	265.07 ± 132.57	272.19 ± 273.26	412.40 ± 339.01^*∗∗*^
Total testosterone (nmol/L)	1.18 ± 0.38	2.39 ± 0.66^b^	2.32 ± 0.69
Androstenedione (nmol/L)	5.76 ± 2.13	11.70 ± 4.64^b^	12.53 ± 4.36
Fasting insulin (pmol/L)	52.08 ± 15.63	66.25 ± 31.60^a^	61.60 ± 24.86
HOMA-IR	1.50 ± 0.19	1.99 ± 1.03^b^	1.79 ± 0.73
AUC-insulin (pmol/L × 120 min)	40884.37 ± 14743.89	55110.14 ± 33112.92^a^	49892.78 ± 22061.11

Baseline parameters were compared with those of a control group (*N* = 30) without PCOS. Age and BMI of PCOS women were calculated on 71 women, while laboratory parameters were available both before and after the treatment in 40 patients. All data are reported as the mean ± SD. ^a^*p* < 0.05 vs. control; ^b^*p* < 0.001 vs. control; ^*∗*^*p* < 0.05 vs. baseline; ^*∗∗*^*p* < 0.01 vs. baseline. ALA: *α*-lipoic acid; AUC: area under the curve; BMI: body mass index; FSH: follicle-stimulating hormone; HOMA-IR: homeostasis model assessment of insulin resistance; LH: luteinizing hormone; MI: myo-inositol; PCOS: polycystic ovary syndrome; SD: standard deviation.

**Table 2:** Results of the treatment with ALA plus MI in the patients divided into subgroups according to the presence of IR and of DRs.

		BMI (kg/m^2^)	FSH (mIU/mL)	LH (mIU/mL)	Estradiol (pmol/L)	Total testosterone (ng/mL)	Androstenedione (ng/mL)	Fasting insulin (pmol/L)	HOMA-IR	AUC-insulin (pmol/L × 120 min)
IR	Baseline	31.08 ± 5.81	5.49 ± 1.70	11.08 ± 4.95	253.32 ± 216.13	2.32 ± 0.42	11.49 ± 4.33	108.40 ± 25.21	3.31 ± 0.91	76749.93 ± 44293.96
	6 months	30.25 ± 6.31^*∗*^	4.90 ± 2.79	9.07 ± 6.50	394.89 ± 314.82^*∗*^	2.22 ± 0.55	10.86 ± 4.22	74.72 ± 20.21^*∗∗*^	2.13 ± 0.66^*∗∗*^	61385.76 ± 19343.06

No IR	Baseline	25.74 ± 4.34	7.38 ± 3.30	13.08 ± 7.83	278.47 ± 293.23	2.39 ± 0.73	11.77 ± 4.82	51.74 ± 17.22	1.51 ± 0.57	47095.35 ± 24335.69
	6 months	25.32 ± 3.89	5.30 ± 2.35^*∗∗*^	10.69 ± 6.97	418.28 ± 353.26^*∗*^	2.36 ± 0.73	13.02 ± 4.40	57.01 ± 25.07	1.67 ± 0.73	45636.11 ± 21788.68

With DRs	Baseline	26.47 ± 4.54	7.47 ± 4.64	10.44 ± 4.84	341.99 ± 389.93	2.29 ± 0.76	11.42 ± 1.86	63.89 ± 29.58	1.85 ± 0.89	64158.06 ± 49596.88
	6 months	25.61 ± 4.14^*∗*^	5.61 ± 2.33	11.22 ± 7.40	440.85 ± 375.32	2.29 ± 0.73	13.55 ± 4.85	61.53 ± 20.63	1.80 ± 0.64	49730.97 ± 19476.60

Without DRs	Baseline	27.27 ± 5.50	6.52 ± 1.64	13.70 ± 8.03	232.73 ± 177.07	2.43 ± 0.62	11.87 ± 3.42	67.43 ± 33.06	2.05 ± 1.10	50767.08 ± 21370.14
	6 months	26.91 ± 5.37	4.95 ± 2.52^*∗*^	9.74 ± 6.56^*∗*^	396.36 ± 324.47^*∗∗*^	2.36 ± 0.66	12.01 ± 4.12	61.60 ± 27.15	1.78 ± 0.78^a^	49970.42 ± 23582.92

All data are reported as the mean ± SD. 17 women had IR, and 49 did not. Women with IR showed a reduction of BMI, fasting insulin, and HOMA-IR and an increase in estradiol, while women without IR showed an increase in estradiol and a reduction of FSH. 26 women had DRs, and 45 did not. Women with DRs only showed a significant reduction of BMI, while women without DRs showed a significant reduction of FSH and LH and an increase of estradiol, with a tendency to a reduction of HOMA-IR. ^*∗*^*p* < 0.05 vs. baseline; ^*∗∗*^*p* < 0.01 vs. baseline; ^a^*p*=0.052. ALA: *α*-lipoic acid; AUC: area under the curve; BMI: body mass index; DRs: diabetic relatives; FSH: follicle-stimulating hormone; HOMA-IR: homeostasis model assessment of insulin resistance; IR: insulin resistance; LH: luteinizing hormone; MI: myo-inositol; PCOS: polycystic ovary syndrome; SD: standard deviation.

**Table 3:** Results of the treatment in two subgroups of women taking the same dose of ALA (800 mg) and two different doses of MI per day (group A 2000 mg; group B 1000 mg).

	Group A		Group B	
	Baseline	6 months	Baseline	6 months
BMI (kg/m^2^)	27.10 ± 4.19	25.02 ± 4.03^*∗∗*^	26.79 ± 6.43	27.15 ± 6.12.62
FSH (mIU/mL)	5.73 ± 1.85	4.88 ± 1.45	7.11 ± 3.23	5.26 ± 2.62^*∗∗*^
LH (mIU/mL)	14.73 ± 8.42	13.42 ± 6.32	12.09 ± 6.95	9.59 ± 6.80^*∗*^
Estradiol (pmol/L)	255.16 ± 219.14	402.01 ± 339.38^*∗*^	277.04 ± 290.22	415.38 ± 345.11^*∗*^
Total testosterone (ng/mL)	2.88 ± 0.73	2.70 ± 0.80	2.18 ± 0.55	2.18 ± 0.59
Fasting insulin (pmol/L)	66.81 ± 30.00	71.04 ± 29.17	61.60 ± 24.86	55.63 ± 20.21
HOMA-IR	1.93 ± 0.91	2.00 ± 0.84	1.79 ± 0.73	1.66 ± 0.64
AUC-insulin (pmol/L × 120 min)	60833.82 ± 19899.02	51441.04 ± 22941.46^*∗*^	52009.79 ± 38484.58	49054.10 ± 22024.79

The higher dose of MI caused changes in BMI, estradiol levels, and AUC-insulin, while the lowest dose caused changes in FSH, LH, and estradiol levels. All data are reported as the mean ± SD. ^*∗*^*p* < 0.05 vs. baseline of the same group; ^*∗∗*^*p* < 0.01 vs. baseline of the same group. ALA: *α*-lipoic acid; AUC: area under the curve; BMI: body mass index; FSH: follicle-stimulating hormone; HOMA-IR: homeostasis model assessment of insulin resistance; IR: insulin resistance; LH: luteinizing hormone; MI: myo-inositol; SD: standard deviation.

## Data Availability

The clinical data used to support the findings of this study are available from the corresponding author upon request.

## Abbreviations

A: Androstenedione
ALA: *α*-Lipoic acid
AMPK: 5′-Adenosine monophosphate-activated protein kinase
AUC-insulin: Area under the curve of insulin
BMI: Body mass index
CV: Coefficient of variation
DCI: D-chiro-Inositol
DHLA: Dihydro-lipoic acid
DM: Diabetes mellitus
DRs: Diabetic relatives
E_2_: Estradiol
FSH: Follicle-stimulating hormone
GLUT4: Glucose transporter 4
HOMA-IR: Homeostasis model assessment of insulin resistance
IPG: Inositol phosphoglycan
IR: Insulin resistance
LASY: Lipoic acid synthase
LH: Luteinizing hormone
MI: Myo-inositol
NF-*κ*B: Nuclear factor kappa-light-chain-enhancer of activated B cells
OGTT: Oral glucose tolerance test
PCOS: Polycystic ovary syndrome
ROS: Reactive oxygen species
SD: Standard deviation
T: Testosterone
TSH: Thyroid-stimulating hormone.
